# Integrated flood risk assessment of properties and associated population at county scale for Nebraska, USA

**DOI:** 10.1038/s41598-023-45827-4

**Published:** 2023-11-11

**Authors:** Shivendra Srivastava, Tirthankar Roy

**Affiliations:** Department of Civil and Environmental Engineering, University of Nebraska – Lincoln, Lincoln, USA

**Keywords:** Hydrology, Natural hazards

## Abstract

Risk assessment of properties and associated population was conducted for the state of Nebraska, leveraging only open-source datasets. The flood risk framework consisted of interactions among drivers, i.e. hazard, exposure, vulnerability, and response, to assess the risks related to properties and associated populations. To quantify hazard on a county scale, we considered properties at risk of flooding based on a flood score (a higher score represents a greater chance of flooding). Exposure was quantified by considering population density at the county level. We quantified vulnerability under four categories: social, ecological, economic, and health. Response, a relatively newer component in flood risk assessment, was also quantified under three distinct categories: structural, non-structural, and emergency. Overall, we found that counties in eastern Nebraska (Sarpy, Dakota, Wayne, and Adams) have a higher risk of flooding consequences due to more exposure to vulnerable assets such as population and property. The assessment also observed that counties in eastern Nebraska are in the process of improving their flood control measures with dams, levees, and higher insurance coverage that can subdue the risks associated with flooding. The results from this study are anticipated to guide water managers and policymakers in making more effective and locally relevant policies and measures to mitigate flood risks and consequences.

## Introduction

According to the Intergovernmental Panel on Climate Change (IPCC) special report on extreme events, the risk associated with disaster can be defined as the likelihood of events that severely alter the normal functioning of a community or society. Specifically, these hazardous, physical events threaten vulnerable peoples’ social conditions^1^. Risk assessments across the literature prevalently follow the IPCC framework, which quantifies risk as a function of hazard, exposure, and vulnerability^2–4^. However, in a recent study, response was included as a fourth component of risk^5^. Response refers to the ability to react to a situation and is often excluded as a risk driver. One of the novelties of our study lies in adding a response component to the flood risk framework due to its potential to subdue the adversity of the event.

In the IPCC risk framework, hazard is a function of scale, including the extent and probability of occurrence of a flood event at a given location. Studies have come up with different ways to quantify the hazard associated with flooding, for example, using the Federal Emergency Management Agency’s (FEMA) maps^2^, flood inundation maps^3^, climatic projections to account for risk associated with climate change^4^, and peak discharge for different flood return periods (1000, 100, and 50 years)^6^. The IPCC defines exposure as the presence of people, livelihood, environmental services and resources, infrastructure, and social and cultural aspects the flooding could adversely affect^1^. Different factors can account for exposure, such as population density, housing units, impervious surfaces, and elevation and density of infrastructures^4^. Vulnerability is the propensity or predisposition to be adversely affected by flooding^1^. To assess vulnerability, studies have considered social dimensions such as age, education, ethnicity, gender, and socioeconomic status^2, 4, 7^. We classified vulnerability into social, ecological, economic, and health dimensions, considering multiple variables under each section. Our study is unique in that it adds different dimensions to vulnerability and gives insights to quantify response while assessing the risks associated with flooding across Nebraska on a county scale.

## Study area, methodology, and datasets information

### Study area

**Figure 1:**
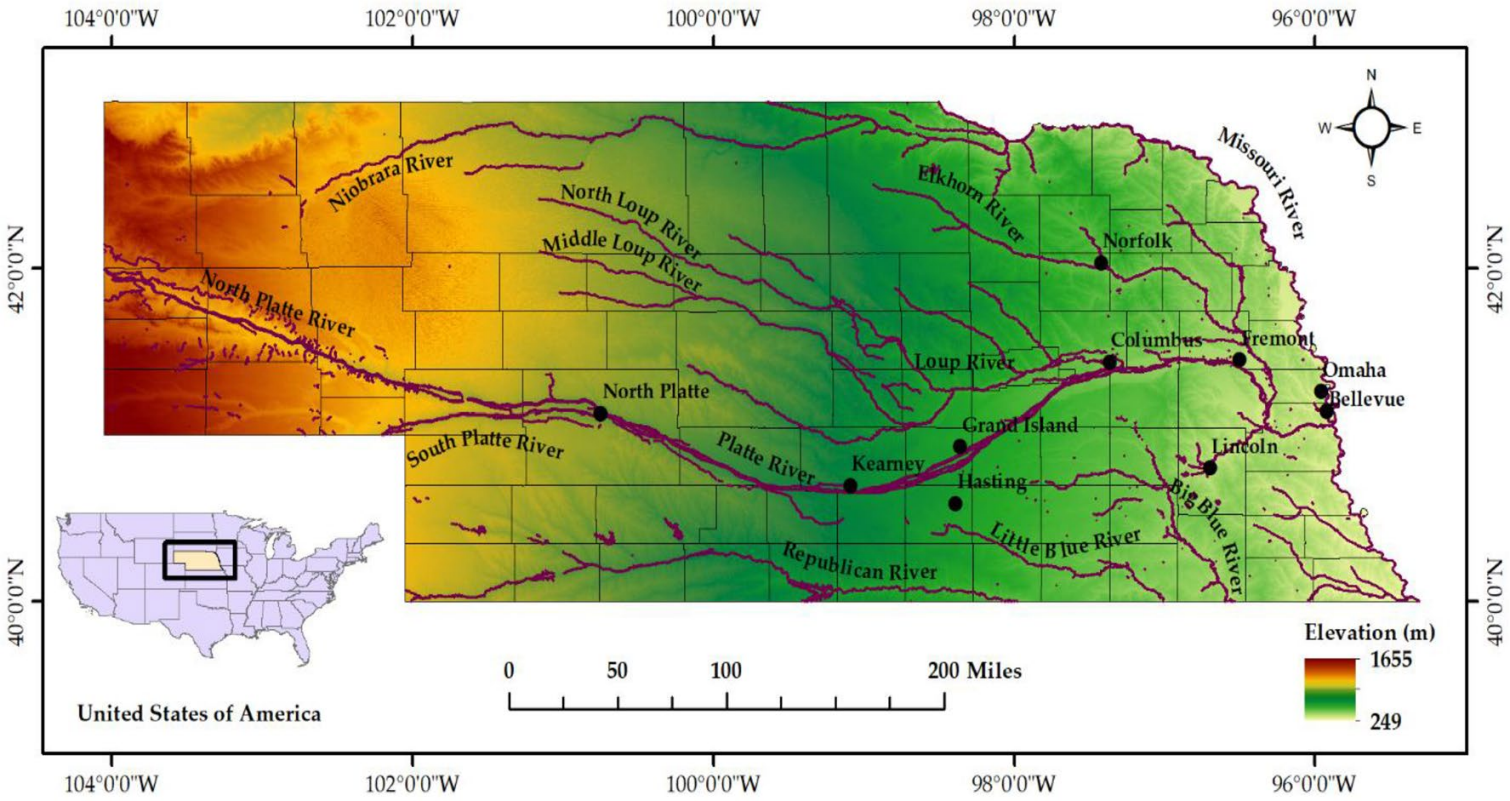
Topographical map of Nebraska. This map was generated using ArcGIS software – version 10.7.1.

Nebraska is a state located in the Midwestern region of the United States. It consists of 93 counties and is surrounded by six states. The topography of Nebraska can be divided into two major land regions – the Dissected Till Plains and the Great Plains (Fig. 1). Dissected Till Plains are located in the eastern part of the state, which consists of rolling hills and fertile agricultural land. This region includes major Nebraska cities – Omaha (Douglas County), Lincoln (Lancaster County), Bellevue (Sarpy County), and Papillion (Sarpy County) (based on existing population). The Great Plains region of Nebraska consists of rolling terrain suitable for agricultural operations. This region is mostly dedicated to corn and wheat fields. The Great Plains of Sand Hills are located in the north-central part of the state, mainly covered with grassy dunes.

Nebraska’s climate and topography play a crucial role when flooding occurs in the state. The Midwestern United States is susceptible to weather patterns, including heavy rainfall, snowstorms, and tornadoes. The region's topography primarily consists of rolling terrain, which makes it even more prone to flooding, as there are no significant natural barriers to counter peak flows. The region also has many major river systems, including the Missouri River, Platte River, and Niobrara River. These rivers and their tributaries drain a large portion of the state and tend to quickly rise and overflow during heavy rain or snowmelt, leading to widespread flooding.

### Methodology

The risk assessment of this study aims to understand and quantify the potential dangers posed by flooding in a comprehensive manner, which can help in making more informed decisions related to effective flood management. The multiplication of the components of risk, i.e., hazard, exposure, and vulnerability is a common approach in risk assessment and has been widely reported in the literature^3, 4, 7^. The multiplication reflects the idea that high exposure to a hazard, combined with high vulnerability, leads to a greater overall risk. Hazard is related to the potential occurrence of a natural or human-induced physical event that can result in loss of life, injury, or other health impacts^1^. Exposure refers to elements such as population, infrastructures, and natural or artificial resources in an area where hazard events may occur. Vulnerability in literature is defined as the propensity to be adversely affected by a disaster event^1^.

In this study, we added one more component, i.e. response, which refers to actions and measures taken to reduce risk^5^. The extent of hazard’s exposure can amplify the risk, especially if that system is highly vulnerable. However, response, in the form of proper actions and measures against flooding, can potentially reduce the risk. In this study, we multiply hazard, exposure, and vulnerability, and divide that outcome by response (as it subdues the risk) to account for the combined effect of drivers on the risk level (Eq. 1).

1 $$\mathrm{Risk}=\frac{\mathrm{Hazard }\times \mathrm{ Exposure }\times \mathrm{ Vulnerability}}{\mathrm{Response}}.$$

Equation (1) considers risk as a function of hazard, exposure, vulnerability, and response, and reflects the understanding that all components contribute to the overall level of flood risk. We considered different variables under each driver of risk, which involved different units of measurement or scales. To allow for meaningful comparison and combination, the variables used to quantify the drivers of risk were scaled from 0 to 1 using (Eq. 2)^4^:

2 $$X=\left(x-\mathrm{min}(x)\right)/(\mathrm{max}\left(x\right)-\mathrm{min}\left(x\right)).$$

By standardizing the variables, we eliminated the potential bias introduced by the magnitude of the values. We assumed equal importance for each variable within a component. Therefore, the values of each risk driver were combined and divided by the number of drivers used^4^, resulting in an index ranging from 0 to 1 (Eq. 3):

3 $$D(\mathrm{0,1})={\sum }{\mathrm{X}}_{D,i} (\mathrm{0,1})/\mathrm{n},$$

where D is the driver's index, X_.D., i_ is the ith scaled variable, and n is the number of variables. Averaging the variables under each component simplified the calculation and interpretation of flood risk.

### Datasets information

The properties at risk of flood hazard were used as a proxy to quantify the hazard. We obtained this information from the First Street Foundation^8^ for 2020. The First Street Foundation uses a probabilistic flood model, which considers the hazard associated with rainfall, riverine flooding, and coastal surge flooding. The model identifies property boundaries and then uses elevation data to determine the likelihood of water reaching the premises. The model also considers adding infrastructure protection, including dunes, wetlands, seawalls, and pumps. To quantify hazard, we reviewed properties in Nebraska counties and collected flood factor scores ranging from 1 to 10, where 1 refers to the lowest chance of a property flooding and 10 refers to the highest. We only considered properties with scores of 8, 9, and 10, as they have severe consequences on properties and the associated population. The exposure variable referring to population density was obtained from the U.S. Census Bureau^9^ for 2020. It should be noted we also used county properties as a component of the exposure variable, but observations and information on properties were only included during the hazard and hazard index Sects. (“Hazard” and “Hazard index”) to avoid repetition in the exposure Sects. (“Exposure” and “Exposure index”).

We collected demographic and social data from the Census and DataUSA^10^ platform for the year 2020, which were used to quantify vulnerability (Table 1). To quantify response, we collected information from different state organizations such as the Nebraska Department of Natural Resources^11^ (NeDNR), State of Nebraska Flood Hazard Mitigation Plan^12^ (SNFHMP), Nebraska Department of Health and Human Services^13^ (NDDHS), U.S. Army Corps of Engineers^14^ (USACE), and Homeless Shelters Directory^15^, an open-source website, which includes information about different emergency shelters located across the state at the county level for 2022 as we did not have information for 2020 (Table 2).

**Table 1:** Variables considered for quantifying vulnerability.

Drivers	Source	Explanation
Social vulnerability		
Age	Census	Percentage of population age under 5 or above 65
Gender	Census	Percentage of female population
Social differences	Census	Percentage of Hispanic, African/black, and Asian population
Ecological vulnerability		
Air pollution	DataUSA	Average daily density of fine particulate matter in micrograms per cubic meter (PM2.5)
Food environment index (FEI)	DataUSA	Index of factors that contribute to a healthy food environment, 0 (worst) to 10 (best)
Occupational hazard	Census	Percentage of population working in the natural resources and transportation sector
Economic vulnerability		
Unemployment	DataUSA	Percentage of population ages 16 and older unemployed but seeking work
Severe housing problem	DataUSA	Percentage of households with at least 1 of 4 housing problems: overcrowding, high housing costs, lack of kitchen, or plumbing facilities
Income inequality	DataUSA	Ratio of household income at the 80th percentile to income at the 20th percentile
Health vulnerability		
Adult obesity	DataUSA	Percentage of adults that report a BMI of 30 or more
Adult smoking	DataUSA	Percentage of adults who are current smokers
Excessive drinking	DataUSA	Percentage of adults reporting binge or heavy drinking
Physical inactivity	DataUSA	Percentage of adults aged 20 and over reporting no leisure-time physical activity
Food insecurity	DataUSA	Percentage of population who lack adequate access to food
Disability	Census	Percentage of Population reported with disability

**Table 2:** Variables considered for quantifying response.

Drivers	Source	Explanation
Structural response		
Number of existing dams	NeDNR	Number of dams located (per sq. mile)
Drainage area of dams	NeDNR	Total drainage area for dams (per sq. mile)
Numbers of existing levees	USACE	Levees in place (length per sq. mile)
Non-structural response		
Conservation measures	SNFHMP	Wetlands area (per sq. mile)
Insurance coverage	SNFHMP	Properties in values ($) insured under flood insurance (residential and commercial) (per sq. mile)
Emergency response		
EMTs availability	NDDHS	Emergency medical technicians (EMTs) available per person
Emergency shelters	Homeless shelters directory	Emergency shelters available per person

## Flood risk framework

**Figure 2:**
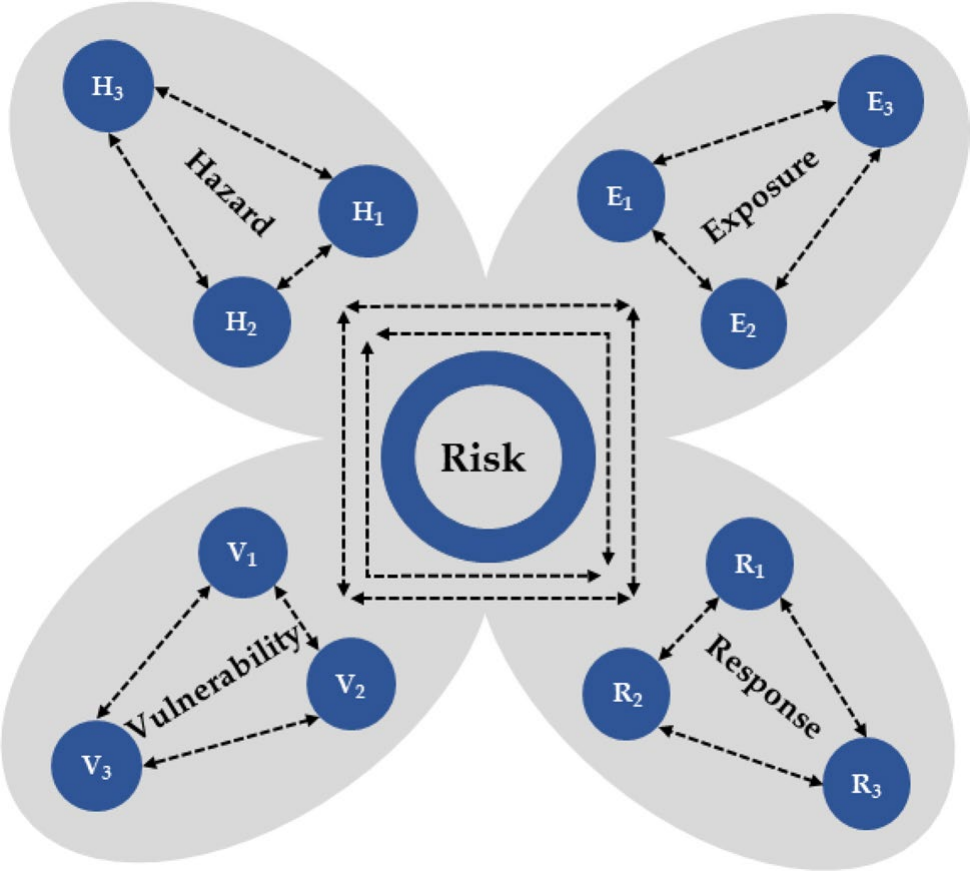
Flood risk framework showing interaction among variables under each driver. These drivers interact with each other resulting in risk.

The risk associated with flooding signifies the possibility of adverse effects on humans and surroundings after the occurrence of flooding events. This risk is derived from the interaction of social and environmental processes, the combination of flood hazards, and the vulnerabilities of exposed elements^1^. Most studies across the literature used the IPCC’s risk framework to estimate risk, i.e. risk as the function of hazard, exposure, and vulnerability. We considered response as the fourth driver of risk (Fig. 2). The response has the ability to reduce the risk of flooding and is required to be included in the existing flood risk frameworks to carry out an effective assessment.

### Hazard

Hazard is considered the potential occurrence of a natural or human-induced physical event that can result in loss of life, injury, or other health impacts^1^. The quantification of flood hazards is carried out at a local and global scale. On a basin scale, one way to quantify hazard is by estimating peak discharge for different flood return periods through statistical modeling and direct observation^6^. On a global or country scale, studies have used climate and flood model outputs^16^. In this study, we considered the number of properties at risk of flooding obtained from flood models to quantify the hazard. The First Street Foundation flood model is a probabilistic flood model considering hazards associated with rainfall, riverine flooding, and coastal surge flooding. The model identifies property boundaries and uses elevation data to determine the likelihood of water reaching properties. It considers community protection and land features such as dunes, wetlands, seawalls, and pumps while quantifying flooding hazards. To assess flood hazards, we considered a flood factor score of 8, 9, and 10, based on the probability of flooding in the area.

### Exposure

Exposure refers to elements such as population, infrastructures, and natural or artificial resources in an area where hazard events may occur. The concentration of the population increases the exposure to extreme events^4^. In the case of flooding, many studies have used population to calculate the risk of exposure^4, 17, 18^. Further, as the number of flooding events is projected to rise, the number of housing units at risk associated with flooding across the United States is likely to triple by the 2050s^19^. Consequently, a larger risk for housing infrastructure will increase maintenance costs, influence public health, and profoundly disrupt struggling families. In this study, housing exposure was also considered, though the information was only included under the hazard component as all properties and their associated risks have already been recorded there.

### Vulnerability

**Figure 3:**
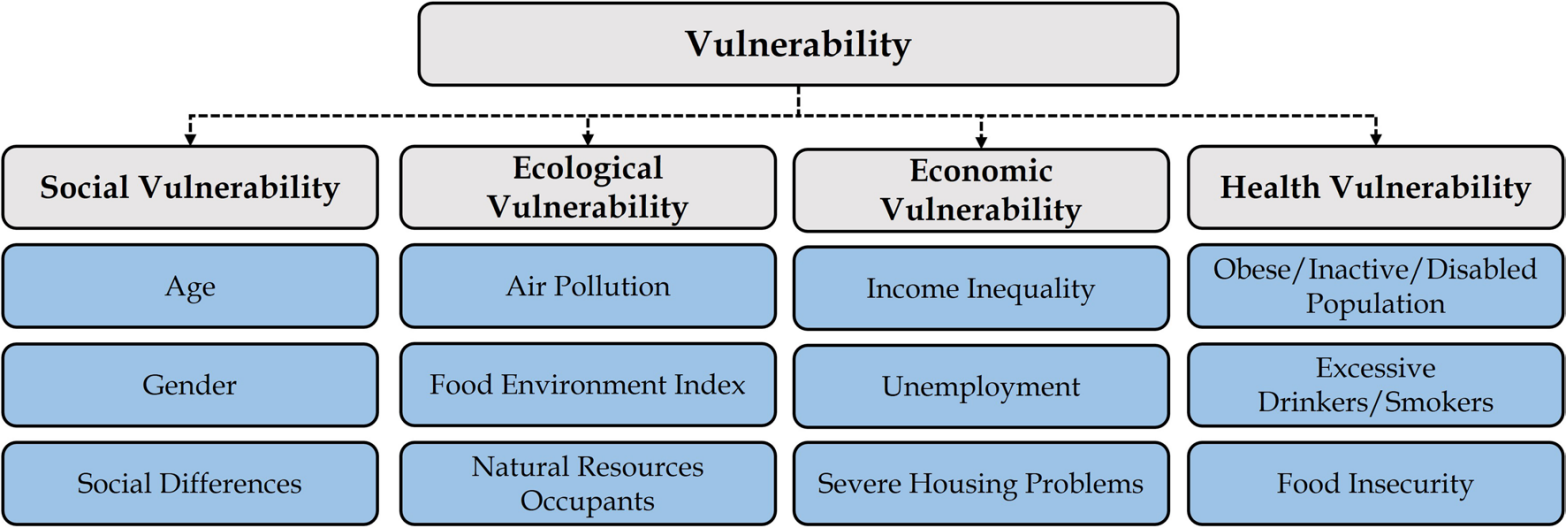
We classified vulnerability under four themes. All variables under each theme were considered for quantification of vulnerability at the county scale.

Vulnerability in literature is defined as the propensity to be adversely affected by a disaster event^1^. Vulnerability indicators are often single variables, though they indicate multidimensional factors such as historical, cultural, social, and economic processes that affect the community’s ability to cope with hazards and respond to them^20^. In this study, we quantified population vulnerability with reference to four themes, i.e. social, ecological, economic, and health (Fig. 3).

#### Social vulnerability

Social vulnerability refers to socio-demographic factors like age, gender, and ethnicity that affect the population’s resilience against flooding. A socially vulnerable population is more likely to be adversely affected during flooding and can take longer to recover^21^. This type of vulnerability plays a crucial role in flooding evacuation processes. For instance, it is more difficult for older people to move to a safe place in the event of a hazard. In some cases, health complications can increase during movement, worsening the situation^2^. The infant population can be considered vulnerable as they require more attention, especially in post-flooding scenarios when hospitals and daycare facilities are affected^3^. Gender can indicate vulnerability due to a lack of resources and differential exposure^2^. Literature has found that women have higher risk perceptions, demonstrate higher preparedness planning, and are more likely to respond to warnings than men; however, in some cases, they are more likely than men to be single parents or primary caregivers to families. Literature has also reported that in many cases, females have relatively lower income, resources, and autonomy than males, which makes them more vulnerable^7, 22, 23^. Race and ethnicity factors are also essential considerations when assessing vulnerability. Ethnic inequality associated with language and cultural barriers, as in the case of immigrants, may hamper flood preparation and evacuation^3^.

#### Ecological vulnerability

Ecological vulnerability can be defined as changes in climatic and environmental conditions that trigger or facilitate other adverse impacts besides flooding. It is difficult to quantify as ecological systems are complex and interconnected, with multiple species, habitats, and environmental processes interacting with each other. Literature has considered different proxies, including proximity of the ecosystem to toxic release inventory and super fund sites, type of existing slope, land use and soil, and availability of green space in dense regions^24, 25^.

Air pollution is one potential quantifying factor. During flooding, it is seen that when a hazardous chemical comes in contact with air, it creates a toxic environment^26^. Fine particulate matter causing air pollution is often attributed to adverse health outcomes^27^. Further, food environment factors, such as accessibility to resources, quality of food available, and price and availability of a product were also considered when assessing ecological vulnerability. Characteristics such as existing biodiversity, access to fresh water and land resources, and overall ecosystem health in a region influence various aspects of food production, distribution, and waste management, which directly impact the food environment index. Ecologically conscious practices can contribute to a healthier, more sustainable, and resilient food environment.

Apart from the drivers of ecological vulnerability mentioned above, literature has also included the effect of habitat disruption, which leads to the loss of food sources for various species, breeding grounds, and nesting sites. These changes disrupt ecological balances, thus impacting biodiversity and causing a rise in health-related issues for human, plant, and animal species^24, 28, 29^. Flooding also alters fish movement, which affects fishing operations^30^, and damaging the forest ecosystem leads to ecological and economical deficits^31^. Therefore, farming, fishing, and forestry are among the primary sectors susceptible to flooding.

#### Economic vulnerability

Economic vulnerability can be defined as the degree to which individuals with low economic status are susceptible to or unable to cope with the adverse effect of flooding. Economic vulnerability is often considered a function of wealth and income^32^. Wealth is an essential factor to consider while assessing people's vulnerability to flooding. Those with low income are more susceptible due to a lack of resources, poor housing, and the inability to recover quickly^2^. Higher wealth increases the possibility of preparing for disaster and leads to a quicker recovery after the event^3^. Literature has also reported that a low economic status results in the prevalence of ill health and societal issues, such as violence, lack of trust, and poor educational facilities, which can be considered a source of economic vulnerability^33^.

Several studies have shown that flooding leads to job loss, financial crises, and adverse health outcomes^34–36^. Similarly, during pre-flooding, the lack of work can be a potential vulnerability factor due to financial constraints. Lack of housing facilities and insurance coverage also results in vulnerability. The population lacking appropriate housing facilities are often victims of intense flood events. Penning-Rowsell^36^ found that a lack of proper kitchen and plumbing facilities, overcrowding, and high maintenance costs increase the risk^37^. The authors also found lack of medical insurance results in unexpectedly high medical costs, increasing the population's financial vulnerability.

It is important to note that the quantification of economic vulnerability is complex and depends on several factors. These factors include complex interdependencies between critical interconnected sectors such as infrastructure, agriculture, industry, and economic services and elements^38, 39^. Assessing the economic losses across these sectors can be challenging. It requires accurate and comprehensive information on financial assets, infrastructure, and economic activities, which may be limited, especially when quantifying at the state or country level. In such conditions, using different proxies to include various aspects can be one of the ways to quantify economic vulnerability.

#### Health vulnerability

Health vulnerability during floods can be defined as the degree to which individuals with health concerns are susceptible or unable to cope with the adverse effect of flooding. Health concerns such as obesity, smoking and drinking habits, and physical inactivity are essential factors that must be considered to quantify the population's vulnerability. Studies have found that managing a patient with morbid obesity needs resources such as personnel, supplies, and specialist equipment^40, 41^. This leads to unique challenges during rescuing and evacuation situations during flooding, and some situations have led to worse conditions^40–42^.

Smoking and drinking habits severely impact human health and increase vulnerability. Smoking rates are higher in populations with post-traumatic stress disorder and are often related to anxiety and depression^43, 44^. Such a population can be vulnerable during flooding, and the situation can heighten their feelings of anxiety and depression. Similarly, many studies have confirmed that the consumption of alcohol among the population increases after a flood event due to higher anxiety and stress^44, 45^, while also being a vulnerability due to its mental impacts^46^.

Research has also found a strong relationship between a population's likelihood of participating in physical activities, such as exercising and cognitive functioning, and flood vulnerability^47^. It is seen that a lack of physical activity results in more stress and agitation, which can hinder crucial cognitive functions during flooding, such as task switching, attention, and proactive response to a situation. Likewise, food shortages can be a potential factor in adverse health outcomes^48^. Studies have found that there is a strong association between food insecurity and adverse health outcomes such as psychosocial dysfunction among young age groups^49^, vulnerability to chronic diseases^50^, and social problems^51^. Flooding can turn into a fatal situation due to the presence of any of these vulnerability factors.

### Response

**Figure 4:**
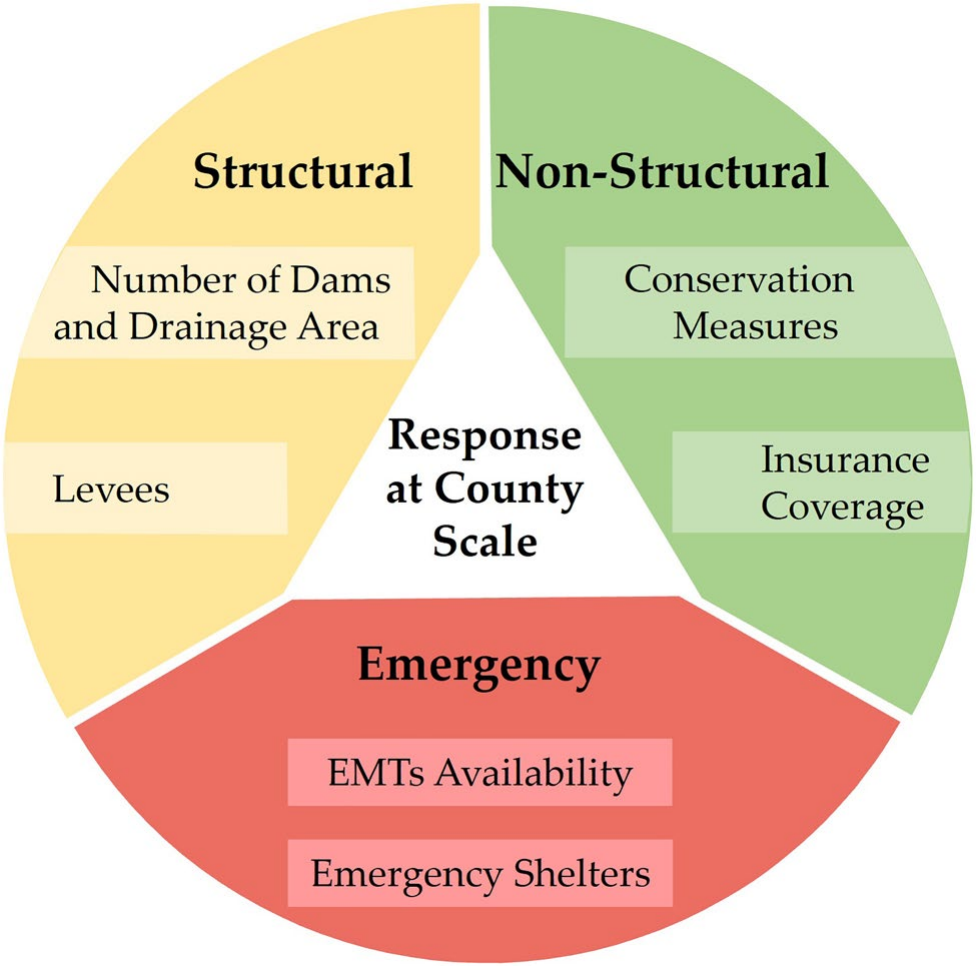
Response component for assessment of flood risk. We considered structural-, non-structural-, and emergency response to quantify response.

Response refers to the ability to react to a situation. It is often excluded as a risk driver but has the potential to subdue the adversity of the event^5^. This component can be considered a link between risk and resilience and can play a vital role in having both positive and negative impacts during flooding. For instance, the displacement of people during a flood can be considered a positive response while also leading to a rise in infectious diseases. Similarly, the construction of flood retaining structures can effectively counter flooding. However, changes in natural landscapes and hydrology can lead to long-term risks associated with natural hazards such as landslides and avalanches. We considered response as a component that will subdue the effect of flooding (Fig. 4).

We divided the response into three major dimensions: structural, non-structural, and emergency. Structural response considers physical infrastructures, which play a crucial role in lowering the intensity of flooding. We considered the number of dams, their drainage area, and levees located on a county scale to account for structural response. Drainage area refers to a land area that contributes water to the dam. The drainage area is significant because it determines the amount of water that can possibly flow into the dam, influencing its water storage capacity and potential for flood control. Consideration of this factor was also important as some Nebraska counties have more than a hundred dams (e.g. Gage, Otoe, and Lancaster), while other counties have a smaller number of dams (e.g. Cedar and Harlan), but they cover more area and have larger storage capacities. This helped in quantifying the actual effectiveness of dams.

A non-structural response is defined as steps that are taken to reduce the ecological and economic impacts of flooding. Non-structural responses include conservation efforts and insurance coverage of properties across the state. Studies have found that implementation of conservation practices such as wetlands can significantly reduce the impact of flooding^52, 53^, which is now part of many conservation efforts carried out at the Natural Resources District level in Nebraska. Likewise, insurance coverage provides financial protection, which overall increases a community's resilience to flooding. Emergency response is an essential aspect in reducing the severity of flood events. We collected information on different emergency shelters and EMTs availability at the county scale and included them as response features. Emergency shelters offer a secure refuge for evacuees, ensuring their immediate safety and basic needs. Meanwhile, EMTs provide critical medical care and triage services, addressing injuries and flood-related illnesses promptly.

## Results and discussion

### Hazard index

Northern and western parts of Nebraska experience flooding due to heavy rainfall, rapid snowmelt, ice jams, and river overflow. This region is home to many rivers, such as the Platte River, Missouri River, Niobrara River, and Elkhorn River (Fig. 1). The Niobrara River, a tributary of the Missouri River, runs through the Sandhill’s region of Nebraska and eventually joins the Missouri River at Niobrara, Nebraska. Further, the Elkhorn River, the tributary of the Platte River, runs through Antelope County and joins the Platte River near Gretna, Nebraska (part of Sarpy County). Additionally, the region’s topography of rolling hills and steep terrain also significantly exacerbates flash flooding in the region^12^.

**Figure 5:**
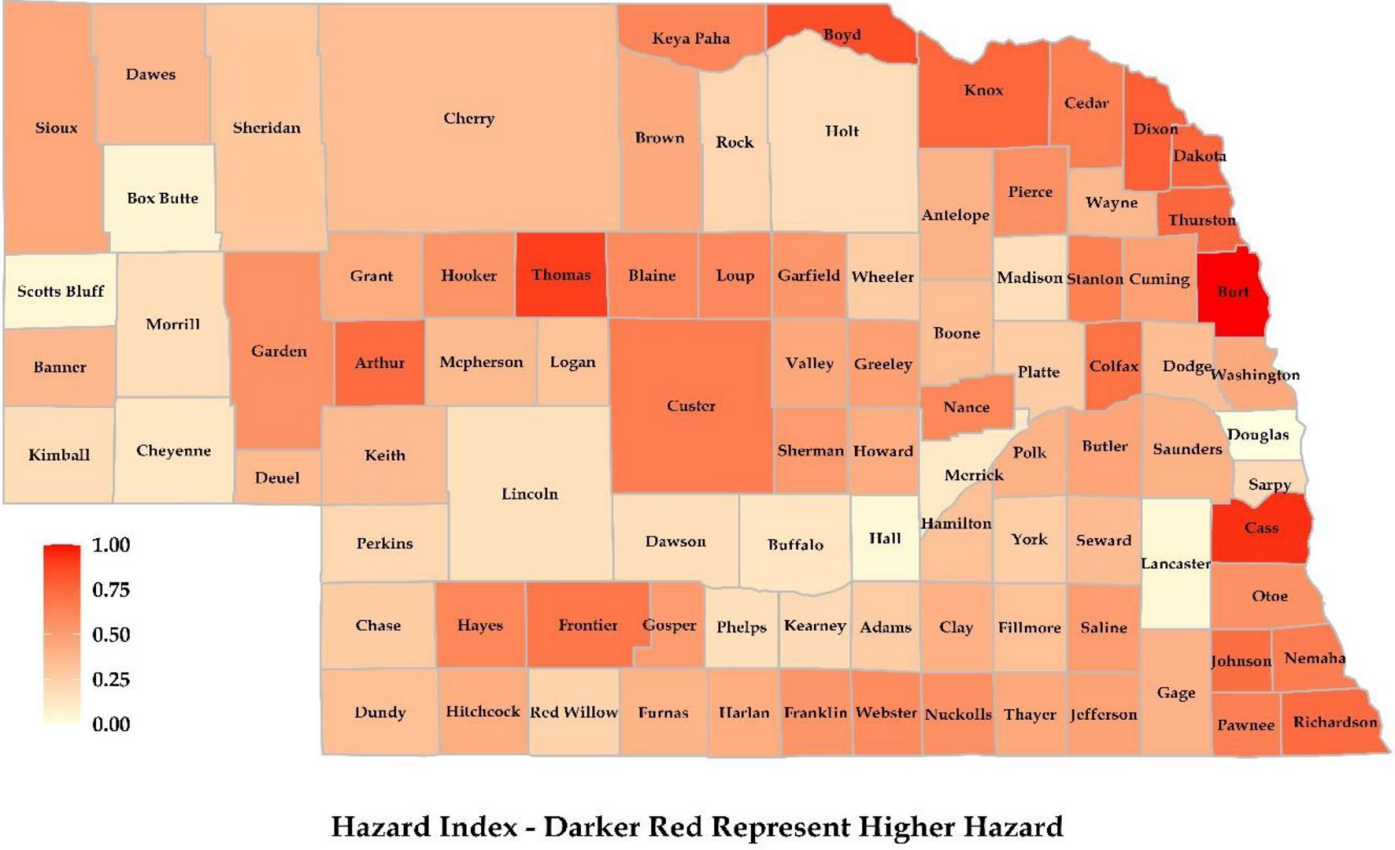
Hazard map was prepared considering properties with flood factors of 8, 9, and 10 at the county scale.

**Figure 6:**
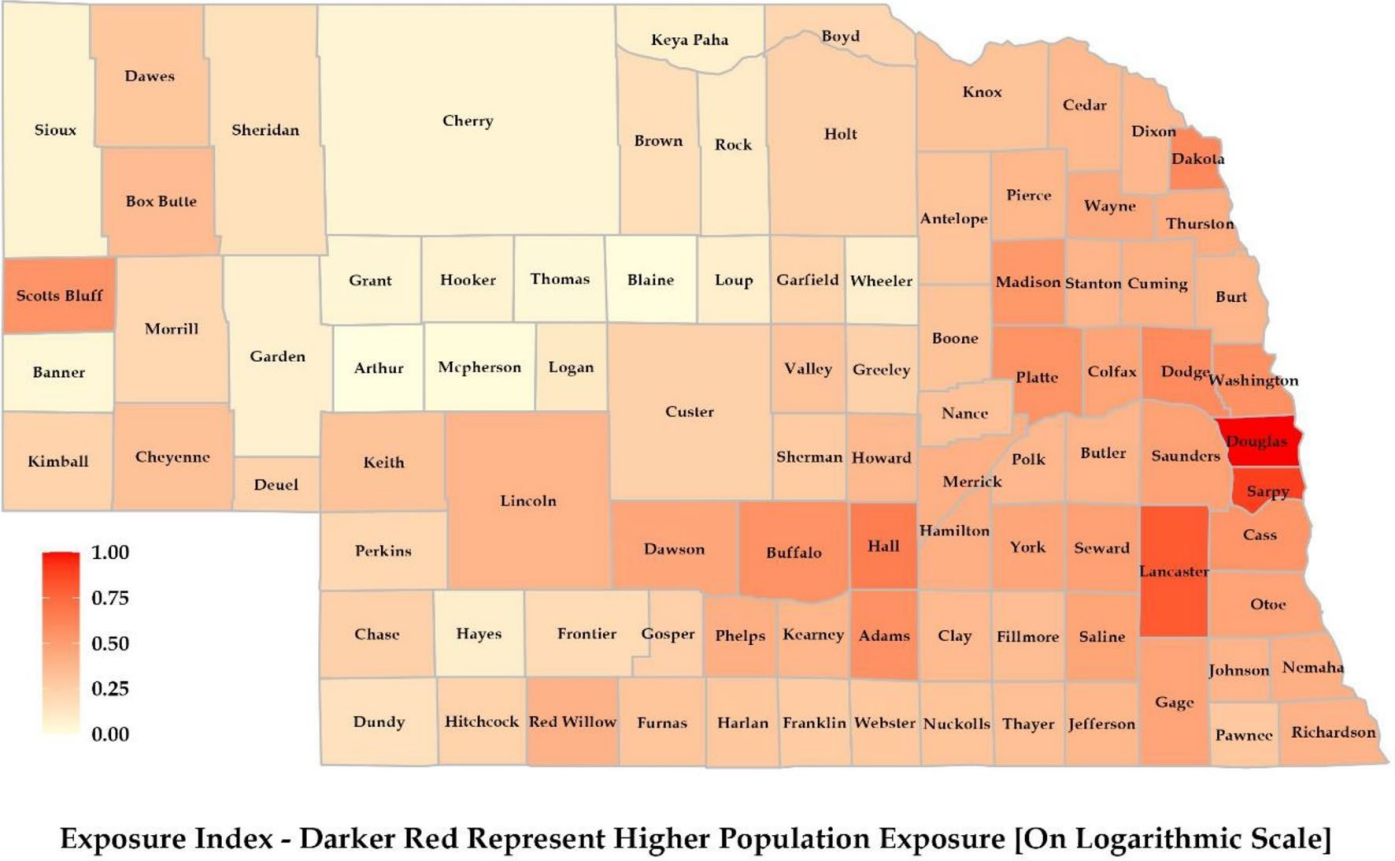
Exposure map was prepared considering population density at the county scale. For representation in map, we transformed population data to logarithmic scale.

**Figure 7:**
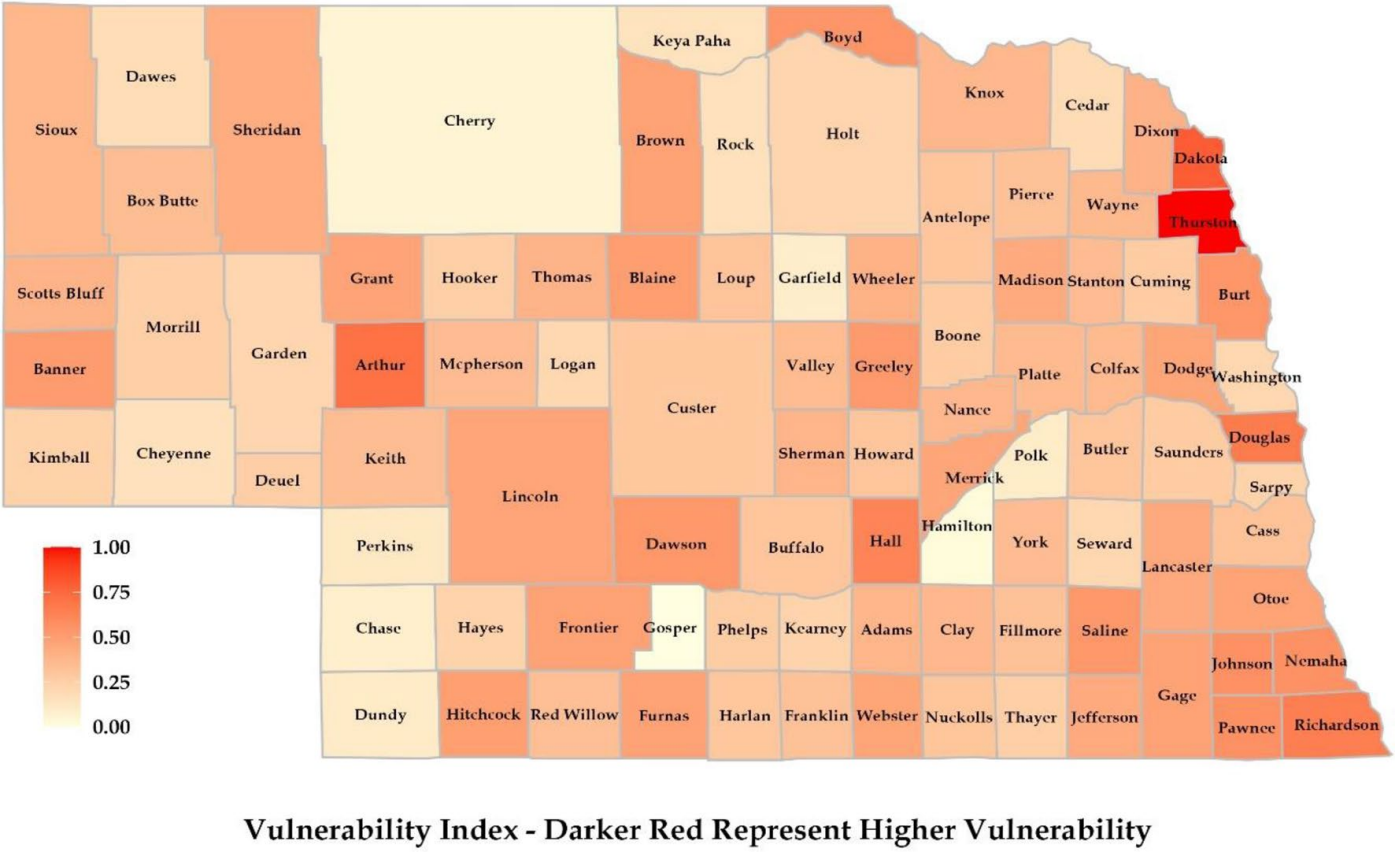
Vulnerability map was prepared considering social, ecological, economic, and health drivers at the county scale.

**Figure 8:**
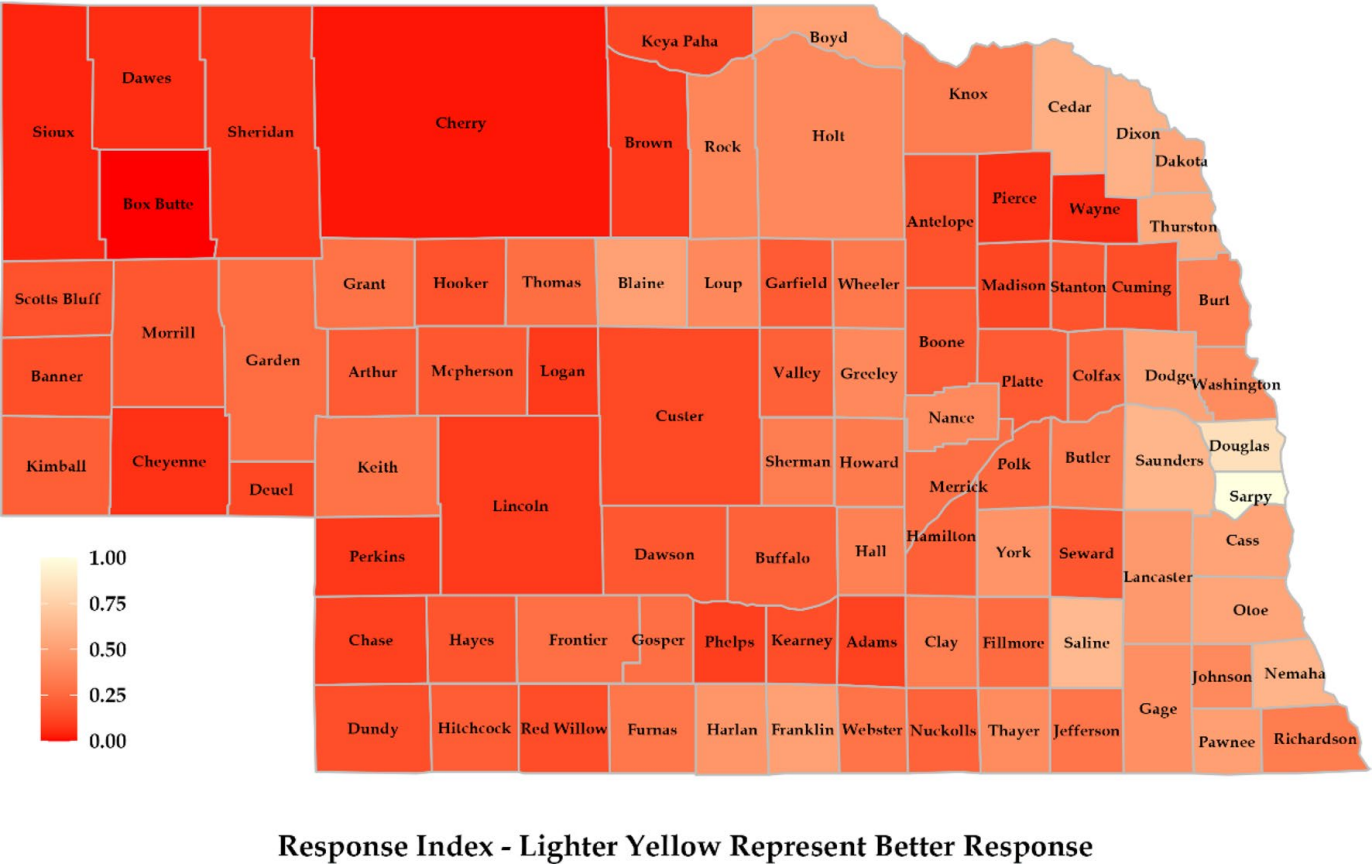
Response map was prepared considering structural-, non-structural-, and emergency response at the county scale.

**Figure 9:**
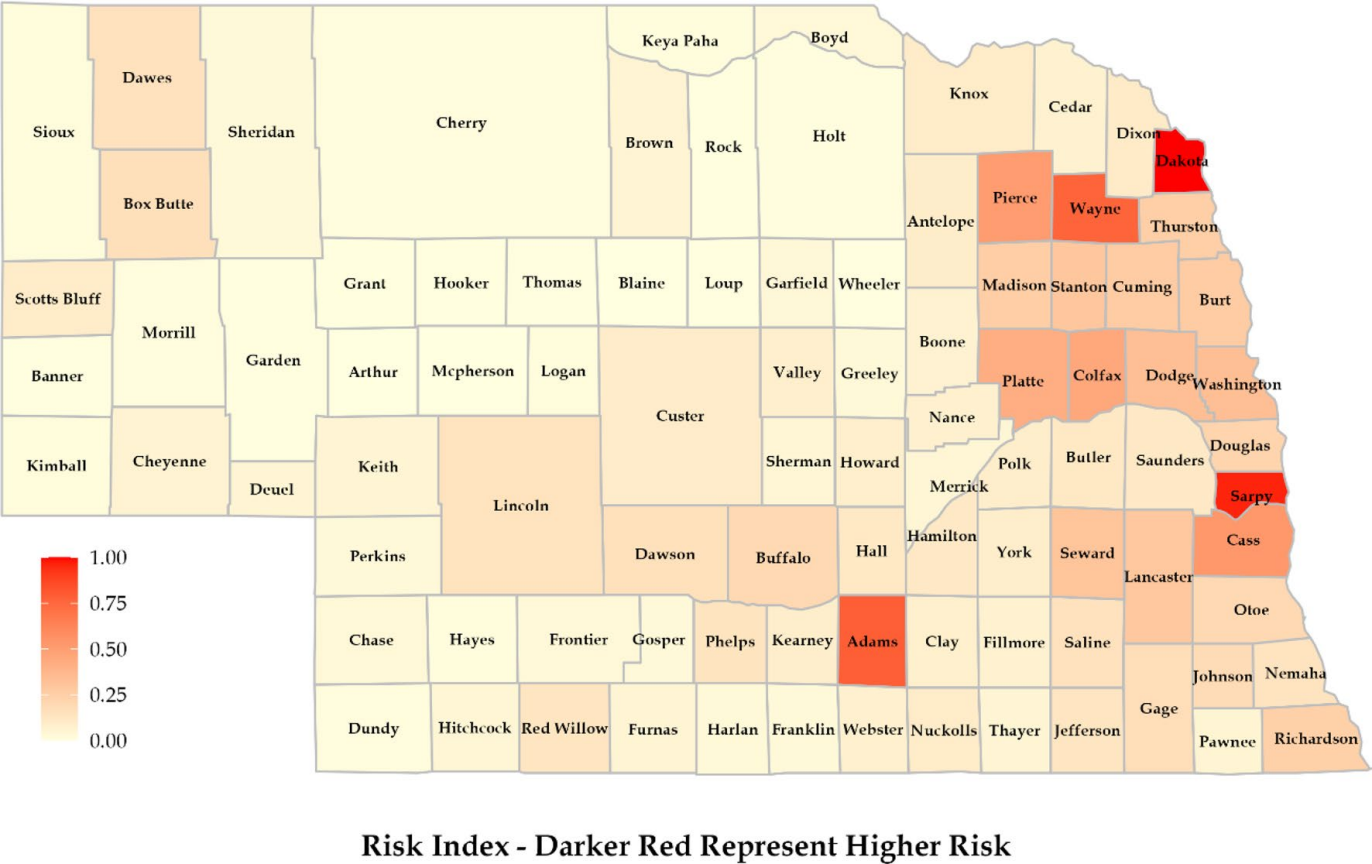
Risk map was prepared considering hazard, exposure, vulnerability, and response at the county scale.

Several counties, including Burt, Boyd, and Cass in the eastern region of Nebraska, showed a higher hazard of riverine flooding (Fig. 5). This can be attributed to the Missouri River, the Platte River, the Elkhorn River, and the Big Blue River, which runs through the eastern counties. Douglas County showed less hazard of property damage since there were fewer properties at hazard relative to the total number of properties, even though it had more properties overall. In the southern region of Nebraska, counties on the eastern front showed a higher flood hazard associated with properties. The higher hazard of property flooding is attributed to the presence of the Republican River, the Big Blue River, and the Platte River, which overflow their banks during periods of heavy precipitation or rapid snowmelt. Flooding in this region is also attributed to existing topography and human activities. Also similar to the northern region, the presence of rolling hills and flat plains, as most of Nebraska’s topography has, increases the chance of flash flooding along with infrastructure development in flood-prone areas. This has significantly altered the natural drainage pattern and increased the likelihood of flooding. Figures 5, 6, 7, 8, 9 were generated using the R-Studio software^54^.

### Exposure index

Northern and Western Nebraska had lower population density, which reduced the overall exposure index of the region (Fig. 6). The majority of the population resides in the urban centers of the state, which are mostly located in the eastern part of the state. Douglas and Lancaster counties had the highest population density exposure. Douglas County has the highest population due to the presence of Omaha, the largest city in Nebraska. Lincoln, situated in Lancaster County and the capital of Nebraska, is the second most populated city. The next highest-populated city is Papillion (Sarpy County), followed by Bellevue (Sarpy County), Grand Island (Hall County), and Kearney (Buffalo County).

### Vulnerability index

In the context of social vulnerability, in Northern and Western Nebraska, counties like Banner, Scotts Bluff, Sioux, and Sheridan showed higher indexes because these regions had a relatively higher proportion of elderly and infant populations (Fig. 7). Language and cultural differences could be observed in these regions due to a substantial number of Hispanic, African/Black, and Asian individuals. On the ecological front, these regions had better air quality (PM 2.5 concentration was the least for Nebraska’s north and western regions). However, factors like lower food environment index and occupational hazard were higher for Grant and Blaine counties, resulting in a higher ecological vulnerability. This part of Nebraska has higher income inequality and less available housing, which resulted in a higher economic vulnerability index. The higher obese and disabled population also increased health vulnerability.

In Eastern Nebraska, Thurston County was found to have the highest health and economic vulnerability, while Douglas, Lancaster, and Dakota showed higher social vulnerability due to the presence of more diverse ethnic groups. In the context of vulnerability, the southern region containing Furnas, Harlan, Franklin, and Pawnee had a higher elderly population, resulting in a higher social vulnerability index. Similarly, Dawson and Hall counties showed higher indexes due to Hispanic and African/Black populations. The other factors which resulted in higher vulnerability included income inequality, food environment index, and the presence of disabled and obese population.

### Response index

The northern and western regions’ response was guided by the presence of more wetlands, which reduces the risk of flooding. Further, the fact that these regions experience less flooding compared to Nebraska’s eastern and southern regions makes them safer and reduces the response requirement (Fig. 8). The regions also showed better response because of better emergency preparation for flooding events. It was observed that emergency shelter availability per person was higher for regions like Blaine, Loup, Thomas, and Grant. However, this can be due to the fact they have a lesser population. In the eastern region, counties like Sarpy, Douglas, Saunders, and Lancaster showed better responses. Counties like Douglas, Sarpy, and Lancaster have higher insurance coverage rates for properties. Also, these regions have more structural flood control measures in the form of levees and dams. However, there have been more flooding events compared to other regions of Nebraska, resulting in higher risk. Response to flooding was well addressed in the southern region. There were several factors behind this. Firstly, a substantial number of dams were located in the area, resulting in higher drainage areas. Secondly, these counties have many natural wetlands, which can effectively reduce the risk of flooding. Third, counties like Dundy, Hitchcock, Red Willow, Franklin, and Webster have seen relatively lower flooding in recent times^12^.

### Risk index

The final risk map showed the overall risk associated with flooding (Fig. 9). This was prepared considering all the drivers (hazard, exposure, vulnerability, and response). Counties located in the eastern part of Nebraska showed higher risk. Dakota and Sarpy showed the highest risk of flooding, while Adams, Wayne, Cass, Pierce, Colfax, and Platte showed a substantial amount of risk. Although counties like Douglas and Lancaster are more densely populated and have a higher density of infrastructures compared to others, they showed relatively lower risk since they have a stronger response capability to flooding. Further, we used the relative value of each variable and scaled them for a better comparative analysis with vastly different population sizes and numbers of housing units. The counties in Western Nebraska showed a lower risk of flooding, which does not imply that no flooding can occur. This part of the state has lower exposure and a less susceptible population, so the chance of severe flooding consequences is reduced. The heightened risk in Dakota County stems primarily from a greater number of properties exposed to hazards, coupled with notable vulnerabilities. Sarpy County’s elevated overall risk, compared to other counties, arises primarily from increased exposure. Additionally, a notable level of vulnerability further elevates the overall risk assessment despite their well-implemented response measures. Also, many counties exhibit varying risk factors across different components. For instance, though Douglas County has more exposure, it has a lower hazard value than other counties such as Adams, Sarpy, Wayne, Cass, Dakota, and Thurston. Overall, these results gave strong insights into flood risk at the county level. They could be a good starting point for other studies to look for flood risk at the county scale while considering an exhaustive list of risk factors.

Introducing response has led to significant changes in the risk map compared to the original risk formulation. Nebraska flood management plan documents the highest frequency of flooding incidents in southeastern counties^12^. These counties include Richardson, Nemaha, Saline, Saunders, Otoe, and Cass, which have endured the substantial impacts of flooding. However, an examination of their response to these flooding challenges reveals that these counties have implemented effective flood control measures. Collectively, they have established over 350 flood-controlling structures, primarily in the form of dams^14^. The same proactive approach applies to their utilization of levees, bolstering their preparedness for flood events^14^. Similarly, counties such as Douglas, Sarpy, Dodge, Lancaster, and Cass have responded by enhancing their property insurance coverage. While the regions in eastern Nebraska remain more susceptible to flooding, these counties have demonstrated noteworthy strategies for mitigating the risk. These types of information are necessary for a more realistic risk assessment. By explicitly considering forms of response, we can gain valuable insights into varying risk profiles, and a better understanding of the overall risk landscape.

### Risk index validation

In 2019, Nebraska experienced one of the deadliest flooding events. Out of 93 counties, 81 declared emergencies with a loss of more than 1.3 billion USD in infrastructure damages^12^. The counties which suffered the most included Adams, Boyd, Buffalo, Colfax, Custer, Dodge, Douglas, Knox, Madison, Nemaha, Pierce, Platte, Richardson, Sarpy, Saunders, Stanton, and Washington. These counties experienced significant damage to population and properties. The loss of infrastructure affected the post-flooding response as it limited access to different areas. Further, these counties were identified as having higher infrastructure damage, crop and livestock losses, evacuations and displaced residents, disruption to services, economic loss, and environmental impacts. Our study also showed substantial risk in most of these areas. However, it should be noted that our analysis focuses on properties and the associated population. The scope can be broadened by adding more information related to economic, environmental, health, and social dimensions.

### Uncertainty sources and future developments

We encompassed different dimensions under each component of risk. To quantify risk, we acquired datasets from various sources such as DataUSA, First Street Foundation, Homeless Shelters Organization, State Organizations, U.S. Army Corps of Engineers database, and U.S. Census Bureau. Note that the quality and consistency of these datasets can vary due to discrepancies in definitions, methodologies, and modes of collection. Future developments can look toward improving data sharing and integration capacity, standardizing data collection methodologies, and conducting collaborations at different levels (researchers, policymakers, and stakeholders).

We explored the risk associated with flooding on a county scale for Nebraska using a unique combination of components and variables. Each driver and the corresponding variable used to quantify them were equally weighted. There can be other ways to come up with the weightage; for instance, Reckien used existing documents and assessment reports for weighing indicators^55^. Bankoff et al. suggested expert knowledge for the selection and weightage of variables^56^, while Rufat et al. utilized the weights developed by prior studies^57^. It would be valuable for future studies to weigh each variable according to their importance in estimating flood risk.

We assessed flood risk associated with properties and population, which can be calculated for other dimensions of floods (geographic, social, and infrastructural). It is difficult to quantify flood hazards at a county or state level at a high resolution due to the computation challenges associated with detailed spatial–temporal modeling. In such cases, studies can work toward developing high-resolution flood maps, which can be used for quantification of flood hazards.

We classified county flood vulnerability into four major types: social, ecological, economic, and health. This allowed us to explore different aspects which affect humankind. There is potential for future studies to expand this list of factors resulting in flood-associated vulnerability. Further, in the context of health vulnerability, more publicly accessible data can be beneficial. Information related to different diseases caused by floods can be helpful in establishing essential risk frameworks.

Quantification of flood-risk response was one of the novelties of this work. We classified response into four classes based on the information obtained from the Department of Natural Resources in Nebraska, the Nebraska Department of Health and Human Services, state flood hazard management plans, and the U.S. Army Corps of Engineers website. It will be essential to develop a system to collect and quantify response information associated with flooding.

## Conclusion

This study gave insights into the flood risk linked to properties and associated population at a county scale for the state of Nebraska. We quantified flood risk by modifying the existing IPCC's risk framework (with hazard, exposure, and vulnerability) to add a response component. We obtained information about properties at risk of flooding, which constituted our hazard component. Exposure was quantified by including population information in the framework. We split the vulnerability factors into four sub-classes to explore how vulnerability can affect flood risk and preparedness. We quantified response to include different mitigation strategies counties are using to counter flooding. The overall flood risk was mostly concentrated in the eastern part of the state, particularly in Sarpy, Dakota, Adams, Wayne, Cass, Pierce, Platte, and Colfax counties. The methodology implemented in this study is not limited to the quantification of flood risk associated with properties. It can be applied to other dimensions of floods and hazardous events like droughts, forest fires, and cold waves. However, the drivers may need to be modified for different cases and hazardous events. A county-scale approach like this will make policymakers aware of the existing risks and vulnerabilities. This can help make tailored, effective, and locally relevant policies at the county level.

## Data Availability

The datasets used and analyzed during the current study are available from the corresponding author upon reasonable request.
